# Childhood hypertension due to a rare cause

**DOI:** 10.1186/1687-9856-2013-S1-P38

**Published:** 2013-10-03

**Authors:** Sachin Mittal, Ameya Joshi, Anitha Ananthan, Jane David, Radha Ghildyal, Tejal lathia, Nikhil Bhagwat, Premlata Varthakavi, Manoj Chadha

**Affiliations:** 1 T.N. Medical College & B.Y.L. Nair Hospital, Mumbai, India

## 

A 3 year 6 month old child, presented with complaints of fever, loose stools and vomiting. The child had no signs of dehydration and was noted to be hypertensive with blood pressure of 120/76mm Hg (95^th^ centile for age, sex and height: 104/65 mm Hg). All the peripheral pulses were equal and well felt. There was no radiofemoral delay, no abdominal bruit. The child had been reared as a female, had normal female external genitalia and no palpable gonads. Urine evaluation, Renalfunction test and USG abdomen were normal. Child had Metabolic alkalosis with hypokalemia. Elevated Urine chloridesuggested a saline non responsive metabolic alkalosis. Markedly elevated 24 hour urine potassium and TTKG (Transtubular potassium gradient) was suggestive of tubular potassium wasting in the presence of hypokalemia.Plasma Renin Activity (PRA) was low, with low normal plasma aldosterone concentration (PAC). Serum Cortisol (8AM) was low with elevated ACTH. Basal and post ACTH 17 hydroxy progesterone was low with elevated total progesterone levels. The karyotype was 46XX.This clinical and biochemical picture was suggestive of 17 alpha hydroxylase (CYP 17) deficiency. The patient was started on hydrocortisone therapy. With this, the blood pressure got controlled, the patient gained height, weight, the pigmentation decreased, serum potassium normalized. Thus, the diagnosis of, 17 alpha hydroxylase deficiency,a rare form of Congenital Adrenal Hyperplasia, should be considered in any child with systemic hypertension, especially in the presence of concomitant hypokalemia with metabolic alkalosis. More commonly, it presents in the peripubertal period, during evaluation of delayed puberty. The presentation as early childhood hypertension is rare. Plan to confirm the diagnosis by genetic analysis.

**Table showing various investigations:** 

Sodium/ Potassium(mEq/l)	147 / 2.4
pH/ HCO3(meq/l)	7.682/ 46.3

pO2/ pCO2 (mmHg)	96/ 38

24 hr urine Potassium(mmol/day)	43

TTKG	8.71

PRA (ng/ml/hr)	0.23(1.31 – 3.59)

PAC(ng/dl)	18.86 (10-70)

Basal cortisol(mcg/dl)	0.77

Plasma ACTH(pg/ml)	140

Progesterone(ng/ml)	7.2 (normal upto 2.3)

17 OH Progesterone(ng/ml)	0.5(0.5- 2.7)

